# Enhancing Patient Understanding of Hospitalization and Post-Discharge Needs: The Impact of Physician-Led Verbal Communication and Teach-Back Method

**DOI:** 10.1007/s11606-025-09510-w

**Published:** 2025-04-23

**Authors:** Payal Parikh, Priya Jaisinghani, Srivarsha Kaloth, Anagha Konakanchi, Naveena Yanamala, Sarang Kim

**Affiliations:** 1 Department of Medicine, Rutgers Robert Wood Johnson Medical School, Piscataway, NJ USA; 2 Department of Medicine, NYU Grossman School of Medicine, New York, NY USA; 3 Rutgers Robert Wood Johnson Medical School, Piscataway, NJ USA; 4 Princeton Day School, Princeton, NJ USA; 5 Division of Cardiovascular Diseases and Hypertension, Rutgers Robert Wood Johnson Medical School, Piscataway, NJ USA

**Keywords:** verbal instructions, teach-back technique, discharge practices

## Abstract

**Background:**

Safe transitions of care post inpatient hospitalization require robust communication practices during the discharge process to ensure patient understanding.

**Objective:**

To determine if physician-led patient communication, inclusive of written and verbal instructions with the teach-back method, improves patient understanding of hospitalization and post-discharge needs.

**Design:**

This pre-post study was piloted at an urban, 600-bed, academic tertiary care hospital over a 3-month period.

**Participants:**

Study participants included adult patients admitted to the hospitalist medical teaching service (MTS).

**Intervention:**

Study participants received both a written summary and verbal instruction reinforced with teach-back method. The instruction included the four core domains of patient education: reason for admission, inpatient management, medication changes, and follow-up plan.

**Main Measures:**

Changes in mean patient understandings scores from pre- to post-intervention were evaluated on a 3-point scale (no, partial, or full understanding) in addition to basic demographics, level of schooling, and primary language spoken to better ascertain the drivers of improved health literacy.

**Key Results:**

Among 120 study participants, mean survey scores in all four testing domains showed improvement in patient understanding of “admitting diagnosis” (21.5%, 95% CI, 0.21 to 0.40; *P* < 0.0001), “treatment undergone” (35.1%, 95% CI, 0.34 to 0.58; *P* < 0.0001), “medication changes” (45.8%, 95% CI, 0.40 to 0.67; *P* < 0.0001), and “discharge follow-up” (38.1%, 95% CI, 0.39 to 0.63; *P* < 0.0001). Mean scores improved more in patients with lower levels of schooling in all testing domains, except for “understanding of medication changes,” showing more improvement in patients with high education achievement (95% CI, 0.08 to 1.09; *P* = 0.027).

**Conclusions:**

While standard discharge practice involves only a printed discharge packet, the use of a written summary of instructions and verbal reinforcement using teach-back methods improves patient understanding and health literacy of post-discharge needs during transitions of care.

**Supplementary Information:**

The online version contains supplementary material available at 10.1007/s11606-025-09510-w.

## INTRODUCTION

A thorough evaluation of discharge practices aimed at augmenting patient understanding is necessary for safe transitions of care. While efforts have centered around improving discharge summaries,^1^ documentation alone is not sufficient to improve health literacy. A successful transition rests on the foundation of physician–patient communication highlighting the core domains of inpatient management, medication changes, discharge instructions, and follow-up plans.^1^ However, patient understanding in these four domains is often shockingly low,^2–4^ and varies among the different domains — inpatient diagnosis, 96%, and post discharge needs, 60%^5^ — and even within domains — 69%, medication dose adjustments, 82% for discontinuations, and 62% for newly prescribed medications — lending to concerns of patient safety.^6^ These findings are most pronounced in older patients and those with lower educational attainment.^7^ Despite this well-established link between limited health literacy and ineffective communication at transitions of care leading to poorer health outcomes and readmissions,^8^ physicians are challenged with the time and resources available to comprehensively delivery discharge instructions.^9^

Robust patient education is associated with lower 30-day readmission rates.^10,11^ More specifically, patients feel more confident in their post-discharge care when education across these four pillars is provided: (1) assistance with medication self-management, (2) patient-centered record ownership, (3) timely follow-up with primary care or specialty care, and (4) a list of “red flags” and how to respond to them.^12^ Furthermore, these educational interventions are instrumental in patients with chronic conditions and cognitive impairment, especially with the inclusion of caregivers.^8,13^

While it is known that patient education is key, the actual communication process remains an area of opportunity. As standard practice across many institutions, nurses complete the discharge process by reviewing a printed packet (several pages) of information which includes a medication reconciliation and post-discharge care instructions, often in English. This packet may include medical jargon and is dependent on the fields filled out by the physician in the electronic medical record. Written instructions alone have proven to be ineffective in augmenting patient understanding. The integration of simplified instructions, written, and/or illustrated material with verbal instruction and utilization of teach-back methods have proven beneficial.^14–17^ However, teach-back, as defined by the patient repeating back the information, seems to be inadequately utilized in the discharge process.^18^

In our 600-bed academic medical center, we evaluated the practice of physician-led patient education with two interventions, a one-page written summary of simplified instructions highlighting the four core domains and a verbal instruction with teach-back to assess for patient understanding. All instructions were provided at the 5^th^ grade reading level, per guidelines for health literacy.^19^ Both interventions were additive to the usual standard of care at our institution: a printed packet of instructions provided by the nurses at discharge. Furthermore, we evaluated patient understanding in the context of primary language spoken and level of schooling completed. We hypothesize that our interventions, augmented by the teach-back method, will further enhance patients’ understanding of their hospitalization and post-discharge care needs.

## METHODS

This pre-post study was piloted at an urban, 600-bed, academic tertiary care hospital beginning in December 2019; however, it ended 3 months later, due to the start of the COVID pandemic in March 2020. The local institutional review board (IRB) reviewed and approved this expedited study.

### Human Ethics and Consent to Participate Declarations

Not applicable.

### Study Participants

All adult patients admitted to the hospitalist medical teaching service (MTS), comprised of faculty attendings and internal medicine residents, were considered eligible for the study. Exclusion criteria included patients that (i) exhibited cognitive impairment and/or (ii) lacked capacity. There were no limitations on language spoken; a translator phone was used for non-English-speaking patients. On day of discharge, the physicians determined if the patient met study criteria and verbal consent was obtained. If patients did not consent to participate, they still received both verbal and written discharge instructions with the teach-back methods; the only difference was that the assessments were not collected. Patients received no compensation for participation.

### Study Design: Training

Physician training was a key component of this study, inclusive of a weekly 30-min training session to standardize the verbal consent process and the elements of the written one-page summary of (1) acknowledging the patient, (2) providing the four elements in a numbered format, and followed by an (3) acknowledgement “we wish you well” statement. Training also included pre- and post-assessment grading, with guidance provided within the Survey Monkey questions as immediate reminders of what constitutes the levels of understanding (Supplementary Table 2).

### Study Design: Interventions

The intervention was administered by the physician providing direct caring for the patient, PGY2/3 (57.5%), PGY1 (25.8%), and attending (16.7%) (Supplementary Table 3). After the one-page summary was written, the verbal consent was administered. After baseline understanding was obtained via the pre-assessment, the patient was given the one-page summary of instructions which served as a script to follow along with the verbal instruction provided by the physician addressing each of the four core domains. During this time, any clarifying questions were answered. Subsequently, the teach-back method was employed to assess understanding in conjunction with the post-intervention assessment, generally about 10–15 min later. If English was not the primary language, a translator phone was utilized. If it was identified that there was still partial or no understanding after the post-assessment, the physician again explained the elements needed to augment patient understanding. The one-page written summary was given to the patient to take home after discharge. This intervention was provided to the patient either before or after the standard nursing workflow of the discharge process.

### Study Design: Pre- and Post-Assessment Grading

The pre- and post-assessments were identical and consisted of four questions addressing the four core domains: (1) “Can you explain the admitting diagnosis to the hospital?” (2) “Can you explain the treatment you underwent during the hospitalization?” (3) “Can you explain any changes that were made to your medications including medications that have been discontinued, dosage changes, and new medication?” (4) “Can you explain your discharge follow up including future doctor appointments?” Responses were scored using the following 3-point scale: 0, “no understanding”; 1, “partial understanding”; and 2, “full understanding.” Guidance on standardized scoring was included in the Survey Monkey next to the questions by way of examples: to grade the admitting diagnosis of congestive heart failure, “no understanding” would be “I don’t know why I am here,” “partial understanding” would be “shortness of breath,” “swelling”; and “full understanding” would be “heart failure” (Supplementary Table 2). The study lead was available to support the physicians administering the intervention. Data was entered in real-time into a secure Survey Monkey account using the provider’s smartphone and no patient identifiers were obtained.

To assess interrater reliability, a subset of patients (*N* = 8) was independently evaluated by a reviewer unfamiliar with the patient’s case (inclusive of 16 random nurses and physicians). This reviewer administered and scored the assessments to compare ratings. The sample size was determined based on pilot study feasibility, the structured nature of the 3-point ordinal scale, and the expected high agreement between raters. While smaller than typical interrater reliability studies, this subset provided a preliminary estimate of agreement while minimizing disruptions to clinical workflow.

### Statistical Analysis

Baseline patient demographics, including age, self-reported gender, race/ethnicity, primary language spoken, and highest level of schooling, were compared among study participants. Univariate comparisons were performed between pre/post-assessment test scores and patient demographics using *t*-test, Chi square, and ANOVA as appropriate. Data from pre- and post-assessment scores, including mean, standard error of the mean (SEM), and score-to-score changes, were summarized. Interrater reliability was assessed using the intraclass correlation coefficient, to estimate the reliability of single ratings and account for response bias. We compared mean patient understanding scores before and after physician verbal and written instructions with a paired *t*-test. Cohen’s *d* was calculated to evaluate the effect size. A repeated measures ANOVA was also performed for comparison and used to verify mean score differences. A within-subject design was employed, where an identical sample of patients was evaluated at the pre- and post-intervention time points.

 All statistical analyses were performed using MedCalc Statistical Software version 22.021 (MedCalc Software bv, Ostend, Belgium). A *P* value of < 0.05 was considered significant.

## RESULTS

### Baseline Characteristics: Characterizing Respondent Demographics

A total 179 patients were approached; 120 patients received the intervention (36 patients did not meet inclusion, 23 patients declined). The population was majority male (61.7%), non-Hispanic White (46.7%), and English-speaking (81.7%). Most participants had completed up to a college degree (40.8%) (Table 1).

**Table 1:** **Baseline Characteristics of Study Participants**

Demographic measure	No. (%)	Female no. (%)	Male no. (%)	*P*-value
Age (SD)	55.7 ± 16.8	55.4 ± 19.8	55.8 ± 14.8	NA
Sex	120 (100)	46 (38.3)	74 (61.7)	< 0.0001
Race				
Asian	21 (17.5)	9 (19.6)	12 (16.2)	0.4475
Black	19 (15.8)	8 (17.4)	11 (14.9)	
White	56 (46.7)	23 (50.0)	33 (44.6)	
Hispanic or Latino	22 (18.3)	5 (10.9)	17 (22.9)	
Other	2 (1.6)	1 (2.2)	1 (1.4)	
Language				
English	98 (81.7)	38 (82.6)	60 (81.1)	0.9748
Other than English	22 (18.4)	8 (17.4)	14 (18.9)	
Highest level of education				
Grades 1–5	4 (3.3)	1 (2.2)	3 (4.1)	0.3208
Grades 6–8	8 (6.7)	2 (4.3)	6 (8.1)	
Grades 9–12	43 (35.8)	17 (37.0)	26 (35.1)	
College	49 (40.8)	23 (50.0)	26 (35.1)	
Post-graduate schooling	12 (10.0)	3 (6.5)	9 (12.2)	
Unsure	4 (3.3)	0 (0.0)	4 (5.4)	

### Pre vs. Post-Intervention Patient Understanding Across the Four Domains

**Figure 1:**
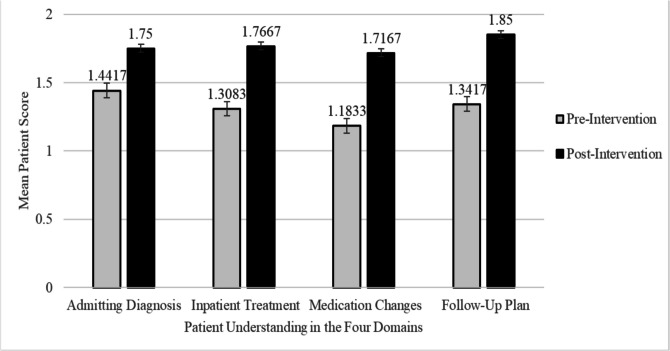
**Pre-post assessment mean score changes.** Pre-to-post assessment mean score changes in the four domains of patient understanding (reason for admission, inpatient treatment, medication changes at the time of discharge, and post-hospitalization follow-up plan) were significantly improved in all four domains (*P* < 0.0001). Error bars indicate standard errors. Scores were evaluated on a 3-point scale of no (1), partial (2), or full understanding (3).

In the pre-post analysis, mean patient understanding scores in all four core domains (reason for admission, inpatient management, medication changes at time of discharge, and post-hospitalization follow-up) showed statistically significant improvement post-intervention (Fig. 1, Supplementary Table 1). For Question 1 (Q1), “Can you explain your admitting diagnosis?”, the average score increased from 1.44 to 1.75 (95% CI, 0.21–0.40; *P* < 0 0.0001). Question 2 (Q2), “Can you explain the treatment?”, average score increased from 1.31 to 1.77 (95% CI, 0.34–0.58; *P* < 0.0001). Question 3 (Q3), “Can you explain medication changes?”, and Question 4 (Q4), “Can you explain your discharge follow-up?”, showed similar improvements, with Question 3 average increasing from 1.18 to 1.72 (95% CI, 0.40–0.67; *P* < 0.0001) and Question 4 average increasing from 1.34 to 1.85 (95% CI, 0.39–0.63; *P* < 0.0001). Cohen’s *d* was calculated to evaluate effect size by relating the mean difference to variability. All testing domains exhibited moderate to a large effect size (Supplementary Table 1).

**Figure 2:**
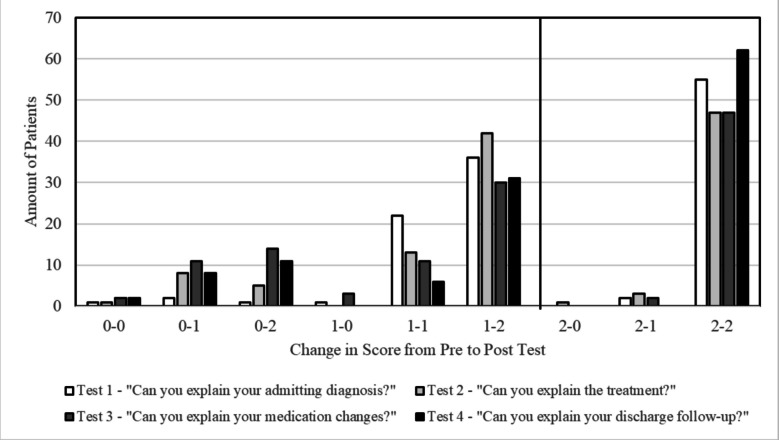
**Score-to-score changes in patient understanding.** This graph depicts pre- to post-assessment score-to-score changes among individual participants in terms of absolute score improvements (0–1, 0–2, 1–2), no change in score (0–0, 1–1, 2–2), and score reductions (2–1, 2–0, 1–0) in the four testing domains. A majority of patients saw no score change, especially among those with pre-assessment full understanding, or improved scores.

Overall patterns of score-to-score changes are shown in Fig. 2. Absolute score improvements (0–1, 0–2, 1–2) were seen for Q1, 32.5%; Q2, 45.8%; Q3, 45.8%; and Q4, 41.7%. Very few patients exhibited a decrease in scores from pre- to post-intervention: Q1, 2.5%; Q2, 3.3%; Q3, 4.2%; Q4, 0%. Many patients had no score changes: Q1, 65%; Q2, 50.8%; Q3, 50%; Q4, 58.3%. However, of those patients seeing no score change, the vast majority had pre-survey scores already indicating full understanding, leaving no room for improvement. Different testing domains also demonstrated variability in pre-survey comprehension, where 47.5%, 42.5%, 40.8%, and 51.7% of patients in Questions 1, 2, 3, and 4 respectively scored for full understanding. Patients with full understanding prior to intervention were unexpected to score lower. Consequently, 45.8%, 39.2%, 39.2%, and 51.6% of patients showed full understanding in pre and post surveys for Questions 1, 2, 3, and 4, respectively.

### Assessing Patient Understanding in Relation to Level of Schooling

When assessing for full patient understanding (score = 2), differences based on education level were noted (Table 2). All four domains showed an increase in mean patient understanding scores across the different levels of schooling completed, with knowledge of medication changes showing the most improvement across all levels of education. Mean scores were most improved in patient groups with the lowest level of schooling completed (i.e., having completed elementary school) for Q1 (0.310, 95% CI, 0.21 to 0.41; *P* < 0.0001), Q2 (0.466, 95% CI, 0.34 to 0.59; *P* < 0.0001), and Q4 (0.509, 95% CI, 0.39 to 0.63; *P* < 0.0001) as compared to those with college or post-graduate completion. In these testing domains, the magnitude of score improvements sequentially decreased with each consecutive higher level of education completed. This trend was seen consistently in all testing domains except for Q3 “medication changes,” which showed more improvement in patients with the highest level of schooling (i.e., post-graduate) (0.583, 95% CI, 0.08 to 1.09; *P* = 0.027).

**Table 2:** **Pre to Post Mean Score Changes as a Function of Schooling Completed**

	Completed Post-graduate schooling	Completed college	Completed high school	Completed middle school	Completed elementary school
**Question 1** Admitting Dx	0.000* (− 0.271 to 0.271)^†^ N/A^‡^	0.197 (0.0579 to 0.336) *P* = 0.006	0.279 (0.176 to 0.382) *P* < 0.0001	0.304 (0.204 to 0.404) *P* < 0.0001	0.310 (0.212 to 0.409) *P* < 0.0001
**Question 2** Hospitalization treatment plan	0.167 (− 0.200 to 0.533) *P* = 0.3388	0.410 (0.252 to 0.568) *P* < 0.0001	0.462 (0.341 to 0.582) *P* < 0.0001	0.464 (0.339 to 0.590) *P* < 0.0001	0.466 (0.343 to 0.588) *P* < 0.0001
**Question 3** Medication* (expert knowledge)	0.583 (0.0795 to 1.087) *P* = 0.0271	0.426 (0.255 to 0.598) *P* < 0.0001	0.510 (0.369 to 0.650) *P* < 0.0001	0.536 (0.398 to 0.673) *P* < 0.0001	0.517 (0.381 to 0.653) *P* < 0.0001
**Question 4** Follow-up	0.333 (0.0205 to 0.646) *P* = 0.0388	0.377 (0.228 to 0.526) *P* < 0.0001	0.462 (0.338 to 0.585) *P* < 0.0001	0.482 (0.362 to 0.603) *P* < 0.0001	0.509 (0.389 to 0.629) *P* < 0.0001

^*^Mean difference

^†^95% CI

^‡^*P*-value

### Patient Understanding Among English and Non-English Speakers

To examine the impact of language on patient understanding, we compared pre- and post-intervention scores between English-speaking (*n* = 98) and non-English-speaking (*n* = 22) participants (Table 3). Non-English speakers demonstrated significantly lower baseline scores in understanding inpatient management (Q2, *P* = 0.011) and medication changes (Q3, *P* = 0.01155). Differences in other domains were not statistically significant. Post-intervention, both groups exhibited improvement across all domains, though non-English speakers continued to have lower absolute scores compared to English speakers.

**Table 3:** **Pre to Post Score to Score Changes as a Function of English and Non-English-Speaking Subjects**

	Non-English speakers (*n* = 22)	English speakers (*n* = 98)	*P* value
	Posttest_Score_2 *n* (%)	Posttest_Score_2 *n* (%)	
Question1_Pretest_Score_0	0 (0%)	1 (1%)	0.638
Question1_Pretest_Score_1	9 (41%)	27 (28%)	0.231
Question2_Pretest_Score_0	3 (14)%	2 (2%)	0.011*
Question2_Pretest_Score_1	9 (41%)	33 (34%)	0.535
Question3_Pretest_Score_0	6 (27%)	8 (8%)	0.012*
Question3_Pretest_Score_1	5 (23%)	25 (26%)	0.770
Question4_Pretest_Score_0	4 (18%)	7 (7%)	0.104
Question4_Pretest_Score_1	5 (23%)	26 (27%)	0.700

### Interrater Reliability

Interrater reliability was assessed on eight patient responses (Table 4). Intraclass correlation coefficients (ICC) showed moderate to high reliability (0.52–0.80) for all items except post-assessment Q1 (0.36). All pre- and post-assessments have strong correlation, except post-assessment Q1 and pre-assessment Q4. Post-assessment Q1 had a correlation coefficient of 0.36 between raters (95% CI, –0.52 to 0.83), indicating a weak relationship, while pre-assessment Q4 had a correlation coefficient of 0.52 (95% CI, –0.32 to 0.88), indicating a moderate relationship. However, all other pre- and post-assessments had correlation coefficients greater than 0.75, indicating strong agreements in scores between raters.

**Table 4:** **Interrater Reliability of Pre/Post Survey Ratings**

Rater 1 vs rater 2	Intraclass correlation (ICC)	95% confidence interval
PreT1	0.7742	0.2680 to 0.9494
PostT1	0.3636	− 0.5212 to 0.8371
PreT2	0.7742	0.2680 to 0.9494
PostT2	1	-
PreT3	0.8000	0.2558 to 0.9570
PostT3	0.7667	0.1672 to 0.9492
PreT4	0.5172	− 0.3202 to 0.8842
PostT4	0.7667	0.1672 to 0.9492

## DISCUSSION

It is well established that poor health literacy leads to poorer health outcomes and readmissions,^8^ especially during transitions of care. This poses an opportunity to incorporate robust physician-led patient education at time of discharge to augment patients’ understanding of both hospitalization and post-discharge needs.^20,21^

Our quality improvement study highlights the implementation of both a one-page written summary at the 5^th^ grade language level, and verbal instructions, with inclusion of teach-back method as part of patient education during the discharge process, especially when both time and resources are limited. Our interventions enhanced immediate recall of the four core domains: (1) reason for admission, (2) inpatient management, (3) medication changes at time of discharge, and (4) post-hospitalization follow-up (Fig. 1). Additionally, our physicians rectified any misunderstanding of the post-discharge instructions in real-time using the teach-back method. In future work, we will incorporate study on how much information was rectified. Our findings expand on existing literature supporting teach-back methods of verbal reinforcement in improving patient satisfaction, post-discharge readmissions, disease self-management, and health-related quality of life.^18,22–24^ Furthermore, in the medication domain, Q3, while only 41% of patients demonstrated full understanding, the lowest of all the pre-assessments, our interventions augmented the largest growth in full understanding in the post-assessment (mean 1.18 – pre, 1.72 – post; 95% CI 0.40 to 0.67, *P* < 0.0001) (Fig. 1). This finding is consistent with limited health literacy for medication changes which contribute to ineffective discharge planning and rehospitalizations^25,28^ but can be mitigated by effective communication strategies such as highlighting key information and ensuring patient comprehension through the “teach-back” method.^26–28^ In the future, incorporation of pharmacist-led interventions may be a value-added intervention.

Lower education levels and language barriers have a direct correlation with limited health literacy at transitions of care affording a significant opportunity for clinicians to tailor their communication to the 5^th^ grade reading level and include translation services.^19,29–31^ In our study, the patients’ level of schooling positively correlated with understanding; notably “full understanding” was the highest in elementary and middle school completions in three domains (Table 2). When comparing the core domains and level of improvement across all levels of school completed, the medication changes domain showed more (> 50%) improvement. Our findings also suggest that non-English speakers had significantly lower baseline understanding regarding inpatient management and medication changes (Table 3). Despite improvement post-intervention, disparities persisted, highlighting the need for enhanced language-access strategies in discharge communication. As medication errors are found in nearly half of patient’s post-discharge, especially with lower numeracy, health literacy, and language barriers,^32,33^ tailoring education to patient appropriate factors is valuable. While our study utilized translator phones, live interpreter involvement and culturally tailored educational resources may further bridge the need. Future research should evaluate the impact of real-time interpreter-assisted teach-back methods to optimize discharge communication in diverse patient populations.

First, limitations on generalizability exist due to the small sample size, at a single institution and a high proportion of college graduates. Furthermore, while schooling and primary language were evaluated, patients with cognitive impairment were excluded, thereby limiting the highest risk group. Further study will focus on a larger sample size and inclusion of patients with cognitive impairment to augment health literacy including that of caregivers. Second, the assessments lend to inherent favorability bias due to the physicians’ familiarity with their own patients. As such, we performed interrater reliability analysis to offset this potential bias and showed that the interrater reliability correlation was highly reliable in most scores (Table 4). Further analysis could focus on inclusion of the bedside nurse to administer the pre- and post-assessment. Third, while our teach-back method enabled immediate recall analysis, we were unable to obtain longitudinal assessments of knowledge retention post discharge.^31^ Our written summary did serve as a take-home document for the patients’ future reference. Fourth, the majority of our patients’ pre-assessment averaged 1, partial understanding, and while there was notable improvement in understanding as demonstrated by a higher partial understanding average in the post-assessment, the 3-point scale limits the true strength of the improvement. We aim to augment the assessment to further delineate the improvement. Lastly, we were unable to assess patient outcomes related to discharge planning, such as readmission rates, due to the early termination of the study. Additionally, this study did not account for hospitalization complexity factors such as length of stay, number of procedures, or number of consultants, which may influence patient understanding. Future research will explore these variables to assess their impact on discharge communication effectiveness.

In the future, we will employ a case control study to evaluate the standard discharge practice compared to the interventions of the written and verbal instruction with teach-back methods. It will include a multifaceted approach with alignment of nursing and physician processes for discharging a patient, use of professional live interpreters, and with long-term assessment of understanding as well as patient outcomes data. In addition, a time and motion study paired with focus groups will adequately assess the feasibility of this intervention in an already busy discharge process.

## CONCLUSION

We increased health literacy of our patients upon discharge by utilizing both a written one-page summary and verbal instructions highlighting the four core domains of admitting diagnosis, inpatient management, medication changes, and post follow-up care; and by ensuring patient understanding with the teach-back method. It is notable that understanding of medication changes during the hospitalization, irrespective of education level, showed the greatest improvement. While most institutions, including ours, continue to rely on only written discharge packets as standard of care, our study highlights that both summarized written instruction and verbal reinforcement using teach-back methods significantly improve patient understanding of post-discharge needs.

## Supplementary Information

Below is the link to the electronic supplementary material.

Supplementary file1 (DOCX 51 KB)
