# Supplementary material for: Enhancing Patient Understanding of Hospitalization and Post-Discharge Needs: The Impact of Physician-Led Verbal Communication and Teach-Back Method
Source: J Gen Intern Med. 2025 Apr 23;40(9):2103–10. doi: 10.1007/s11606-025-09510-w (PMC12325820; doi:10.1007/s11606-025-09510-w)
Supplement: Supplementary file 1 — Supplementary file1 (DOCX 51 KB) [file 11606_2025_9510_MOESM1_ESM.docx]

**APPENDIX**

**Supplementary Table 1: Pre to Post-Intervention Mean Patient Scores**

| **Question** | **Pre** | | **Post** | | **Paired** | | | | |
| --- | --- | --- | --- | --- | --- | --- | --- | --- | --- |
|  | Mean | SE | Mean | SE | Mean | SEM | 95% CI | P-value | Cohen’s d |
| 1 | 1.44 | 0.05 | 1.75 | 0.04 | 0.31 | .049 | 0.21 to 0.40 | <0.0001 | 0.59 |
| 2 | 1.30 | 0.06 | 1.77 | 0.04 | 0.46 | 0.06 | 0.34 to 0.58 | <0.0001 | 0.80 |
| 3 | 1.18 | 0.07 | 1.72 | 0.05 | 0.53 | 0.07 | 0.39 to 0.67 | <0.0001 | 0.80 |
| 4 | 1.34 | 0.07 | 1.85 | 0.04 | 0.51 | 0.06 | 0.39 to 0.63 | <0.0001 | 0.83 |

**Supplementary Table 2: Pre/post-test Survey Questions with Grading Guidance**

| **Testing Domain** | **Survey Question** |
| --- | --- |
| #1 | Can you explain the admitting diagnosis to the hospital?  0: no understanding: patient is unable to describe the presenting problem (i.e "don't know why I am here")  1: partial understanding: patient is able to describe a presenting problem (i.e "shortness of breath, swelling")  2: full understanding: patient is able to provide an admitting diagnosis (i.e "CHF") |
| #2 | Can you explain the treatment you underwent during the hospitalization?  0: no understanding: patient is unable to explain any aspect of the treatment provided (i.e."My wife brought me in")  1: partial understanding: patient is able to explain the treatment provided (i.e "needed to get fluid out")  2: full understanding: patient accurately describes the treatment provided (i.e. "given a diuretic to remove fluid for heart failure") |
| #3 | Can you explain any changes that were made to your medications including medications that have been discontinued, dosage changes, and new medication?  0: no understanding: patient unable to state that there were med changes and unable to show a list  1: partial understanding: patient able to show general understanding of changes that were made to medications and/or are able to show a list of med changes made. (patient can use personal medication list)  2: full understanding: patient able to completely explain all changes to medications including medications that were discontinued, dosage changes, and new medications (patient can use personal medication list) |
| #4 | Can you explain your discharge follow up including future doctor appointments?  0: no understanding: patient unable to state that there were med changes and unable to show a list  1: partial understanding: patient able to show general understanding of changes that were made to medications and/or are able to show a list of med changes made. (patient can use personal medication list)  2: full understanding: patient able to completely explain all changes to medications including medications that were discontinued, dosage changes, and new medications (patient can use personal medication list) |

**Supplementary Table 3: MTS Team and Who Administered Test**

| **MTS Team** | **Total n (%)** | **Female n (%)** | **Male n (%)** | **P-Value** |
| --- | --- | --- | --- | --- |
| A | 27 (22.5%) | 14 (51.9%) | 13 (48.1%) | 0.2203 |
| B | 12 (10.0%) | 2 (16.7%) | 10 (83.3%) |  |
| C | 30 (25.0%) | 9 (30%) | 21 (70%) |  |
| D | 31 (25.8%) | 12 (38.7%) | 19 (61.3%) |  |
| E | 20 (16.7%) | 9 (45%) | 11 (55%) |  |
| **Who administered test** | |  |  | 0.9813 |
| Attending | 20 (16.7%) | 8 (40%) | 12 (60%) |  |
| Resident (PGY2/PGY3) | 69 (57.5%) | 26 (37.7%) | 43 (62.3%) |  |
| Intern (PGY1) | 31 (25.8%) | 12 (38.7%) | 19 (61.3%) |  |
